# Salivary Microbiota Shifts under Sustained Consumption of Oolong Tea in Healthy Adults

**DOI:** 10.3390/nu12040966

**Published:** 2020-03-31

**Authors:** Zhibin Liu, Hongwen Guo, Wen Zhang, Li Ni

**Affiliations:** Institute of Food Science & Technology, Fuzhou University, Fuzhou 350108, China; liuzhibin@fzu.edu.cn (Z.L.); guohongwen@fzu.edu.cn (H.G.); zhangwen@fzu.edu.cn (W.Z.)

**Keywords:** oolong tea, phenolic profile, salivary microbiota, 16S rRNA sequencing, bacterial diversities, correlation network

## Abstract

Tea is the most widely consumed beverages next to water, however little is known about the influence of sustained tea consumption on the oral bacteria of healthy adults. In this study, three oral healthy adults were recruited and instructed to consume 1.0 L of oolong tea infusions (total polyphenol content, 2.83 g/L) daily, for eight weeks. Salivary microbiota pre-, peri-, and post-treatment were fully compared by high-throughput 16S rRNA sequencing and multivariate statistical analysis. It was revealed that oolong tea consumption reduced salivary bacterial diversity and the population of some oral disease related bacteria, such as *Streptococcus* sp., *Prevotella nanceiensis*, *Fusobacterium periodonticum*, *Alloprevotella rava*, and *Prevotella elaninogenica*. Moreover, via correlation network and Venn diagram analyses, seven bacterial taxa, including *Streptococcus* sp. (OTU_1), *Ruminococcaceae* sp. (OTU_33), *Haemophilus* sp. (OTU_696), *Veillonella* spp. (OTU_133 and OTU_23), *Actinomyces odontolyticus* (OTU_42), and *Gemella haemolysans* (OTU_6), were significantly altered after oolong tea consumption, and presented robust strong connections (|r| > 0.9 and *p* < 0.05) with other oral microbiota. These results suggest sustained oolong tea consumption would modulate salivary microbiota and generate potential oral pathogen preventative benefits. Additionally, diverse responses to oolong tea consumption among subjects were also noticed.

## 1. Introduction

An estimation of 700 diverse bacterial species have been identified in human oral cavities, which constitute complex microbial communities [1]. These bacteria generally inhabit at different oral niches, including saliva, supragingival plaque, subgingival plaque, and mucosa. Of these niches, saliva harbors as much as 10^8^ bacteria/mL and constitutes a reservoir of microorganisms regularly derived from dental plaque biofilms adhering to gingival crevices, periodontal pockets, the dorsum of the tongue, and other oral mucosal surfaces [2]. As an integral part of oral microbiota, salivary microbiota has been found to be differentiated between patients with a healthy oral cavity and those with dental caries and periodontitis [3]. Additionally, several studies discovered marked clinical importance of salivary microbiota on the general health of the host, such as by either preventing or causing infections [4]. Thus, salivary microbiota may provide further insight into the integral microbiota structure within the human oral cavity, and even the oral and general health status of individuals.

Since the oral cavity is exposed to the external environment, the salivary microbiota may be influenced by various factors, including oral hygiene, smoking, nutrients, mechanical stress, and the overall health condition of the host [5]. The impact of nutritional factors in shaping the oral microbial ecosystem cannot be ignored. Food residuals in the mouth can be utilized as substrates for oral bacteria; moreover, some food components have a selective effect on microbial growth, by either stimulating or suppressing some specific bacteria. For example, a regular consumption of polyphenol-rich beverages and foods, such as tea, cranberry, coffee, grape, almond, and alcohol-free red wine, have been reported to inhibit oral pathogenic bacteria [6,7,8]. The suppression of oral, especially periodontal pathogenic, bacteria may ameliorate the control of plaque biofilms, and thus reduce the inflammatory and immunological processes of oral and periodontal diseases [9]. Recently, the impact of nutraceutical dietary aliments, such as antioxidants, probiotics, natural agents, and vitamins, on oral health is gaining more and more attention [10].

Tea (*Camellia sinensis*), second only to water, is the most widely consumed beverage in the world. The major constituents of tea leaves are the flavonoids, including flavonols, flavones, and flavan-3-ols, of which over 60% are the flavan-3-ols, commonly referred to as catechins. Based on the United States Department of Agriculture (USDA) Flavonoid Database, it has been estimated that the daily total flavonoid intake is mainly from flavan-3-ols (83.5%); while, the major source of flavonoids is tea (157 mg), and citrus fruit juices come second (8 mg) [11]. There is a large population of heavy tea consumers all over the world, especially in the southern part of China, where people consume a substantial amount of tea infusions on a daily basis. A number of health-promoting effects have been associated with tea consumption; these effects are generally attributed to the phenolic compounds in tea. Tea polyphenols are well known for their antimicrobial properties, including on *Streptococcus mutans* and *lactobacilli* [12], and thus, they are believed to possess anti-cariogenic effects [13,14]. Moreover, regular consumption of tea has proved to exert gut microbiota regulation effect [15,16]. However, with regard to the normal balanced oral microbiota, little is known about the influence of tea drinking. Considering the wide range of biological properties, including anti-microbial, anti-oxidant, anti-inflammatory, anti-cariogenic, and gut microbiota regulation effects of tea polyphenols, it is reasonable to assume that sustained tea drinking will result in certain oral ecological shifts. A better understanding of the oral ecological shifts under sustained and significant tea consumption may contribute to oral health management for tea consumers.

It is also worth noting that, due to the variability in genes, social habits, hormonal fluctuation, diet, quality and quantity of saliva, etc., the oral environment differs between subjects and represents huge inter-individual variations [17]. Moreover, the responses of oral microbiota of different individuals to certain nutritional factors maybe also be diverse. To understand the influence of tea consumption on oral microbiota, tracking the temporal dynamic of salivary microbiota of subjects separately may provide useful information free from interference of inter-individual variations. In the current study, it is hypothesized that sustained tea consumption will alter the composition of salivary microbiota and exert oral health benefits to the host. To test this hypothesis, three orally healthy subjects were recruited and instructed to consume a substantial amount of tea infusions on a daily basis and their salivary bacterial communities pre-, peri-, and post-treatment were quantified by utilizing a high-throughput HiSeq sequencing technique. Then, via several multivariate statistical analyses, the temporal dynamics of salivary microbiota of each individual were analyzed. Based on these, the impact of sustained consumption of tea on the normal balanced oral microbiota was discussed.

## 2. Materials and Methods

### 2.1. Oolong Tea Infusion Preparation and Phenolic Profile Analysis

The tea used in this study was an oolong tea variety, purchased from a local market in Fujian Province, China. The oolong tea was prepared in accordance with the tea consumption method of local residents. A certain amount of dry oolong tea (whole leaves) was immersed in 20 times the volume of distilled boiling water (temperature around 90–95 °C) for 1 min, then the tea leaves were filtered, and the liquor was retained as an oolong tea infusion.

The phenolic profile of the tea infusion was then analyzed by utilizing ultra-high performance liquid chromatography (UHPLC) coupled to quadrupole time-of-flight mass spectrometer (Q-TOF MS/MS) approach, as previously described [15]. Briefly, chromatography separation was performed on an Acquity UHPLC system (Waters, Milford, MA, USA) with HSS T3 column (100 mm × 2.1 mm, 1.7 μm). A sample of 1 μL was injected and eluted with the mobile phase at 0.3 mL/min at 40 °C; detection was at 280 nm. The mobile phase consisted of (A) 0.1% formic acid solution (v/v) and (B) acetonitrile with 0.1% formic acid (v/v), while the gradient program was as follows: 99%–93% (A) in 0–2 min; 93%–60% (A) in 2–13 min; 60%–1% (A) in 13–14 min. The eluent was then introduced to a SYNAPT G2-Si high-definition mass spectrometer (Waters, Milford, MA, USA) equipped with an electrospray ionization (ESI) source. The analyses were performed in negative-ion mode and positive-ion mode, with a sampling cone voltage of 40.0 V, and a capillary voltage of 2500 V. The source temperature was 120 °C, with a desolvation gas flow of 800 L/h at a temperature of 450 °C. The time-of-flight (TOF) acquisition rate was 0.2 s/scan with 0.01 s inter-scan delay. Data were collected in centroid mode from 100 to 1200 Da in full scan during 0–14 min. The mass data were corrected during acquisition using a lock-mass calibrant of leucine enkephalin (200 ng/mL), via a lock spray interface at a flow-rate of 50 μL/min, generating a reference ion for positive ion mode ([M+H]^+^ = 556.2771) and negative ion mode ([M–H]^−^ = 554.2615) to ensure accuracy during the MS analysis. All data analyses were conducted using the MarkerLynx application manager software (version 4.1, Waters, Milford, MA, USA). The total polyphenols content in the tea infusions was then measured by utilizing the Folin–Ciocalteu method [18]. Briefly, 1 mL sample, 5 mL Folin–Ciocalteu’s reagent (diluted 10 times), and 4 mL sodium carbonate (7.5%, w/v) were mixed. After 60 min, the absorbance at 765 nm was measured. Total phenolic content was expressed as a mass percentage on dry matter basis. Gallic acid was used as an external standard.

### 2.2. Subject Enrollment, Study Design, and Salivary Sample Collection

The inclusion criteria for this study included: healthy adult individuals sharing a relatively similar living environment; no tea and antibiotics taken in the previous 3 months; and no smoking. After the screening process, three healthy adult Chinese individuals (2 females and 1 male), 23 years of age, were enrolled from the campus of Fuzhou University, Fuzhou, China. The plaque and gingival status was examined before and after tea intervention. No obvious change was observed either before or after tea usage. In addition, no adverse reaction was reported throughout the experimental period by participants. Written informed consent was obtained from each participant. This study was approved by the ethical committee of the Institute of Food Science and Technology of Fuzhou University (approval number: IFSTFZU20180301).

This study consisted of a 3-day baseline period, an 8-week oolong tea infusion intervention period, and a 4-week follow-up period. During the intervention period, the three subjects (subject 1, subject 2, and subject 3) were required to consume 1.0 L of oolong tea infusion per day (0.5 L in the morning and 0.5 L in the afternoon). Moreover, they were also instructed to circulate or swish the infusion around in their mouths prior to swallowing the tea infusion. During the follow-up period, the subjects were asked not to consume any tea drinks. In addition to this, the subjects were asked to maintain their regular diet and oral hygiene habits, with the exception of the sampling occasions. Salivary samples were collected at 4 different stages, each stage included 3 sequential days: (A) 3 sequential days of the baseline period, which was prior to the intervention period; (B) 3 sequential days after 4 weeks of the tea intervention; (C) 3 sequential days after 8 weeks of the tea intervention; and (D) 3 sequential days at the end of the follow-up period, which accounted for 4 weeks post-intervention. All salivary sample collections were conducted in the morning. Each subject was asked not to eat, drink, or brush their teeth before the sample collection. Then, 2 mL of unstimulated saliva were collected from the subjects by expectoration into a tube. In total, 36 salivary samples from the 3 subjects were sampled.

### 2.3. Salivary Bacterial DNA Extraction

Salivary bacterial DNA was extracted from the 36 salivary samples by utilizing a rapid DNA extraction kit (BioTeke Corporation, Beijing, China), following the manufacturer’s instructions. The extracted bacterial DNA was then checked by agarose gel electrophoresis.

### 2.4. Illumina Sequencing of Salivary Bacteria

Bacterial primers 341-F (5′-CCT AYG GGR BGC ASC AG-3′) and 806-R (5′-GGA CTA CNN GGG TAT CTA AT-3′) with specific barcodes were used to amplify the V3–V4 region of bacterial 16S rRNA genes. The sequencing library of bacterial 16S rRNA genes was generated for high-throughput sequencing, employing the TruSeq^®^ DNA PCR-Free Sample Preparation Kit (Illumina, San Diego, CA, USA). Next, the library was sequenced on an Illumina HiSeq2500 platform by Novogene Bioinformatics Technology Co., Ltd. (Beijing, China).

### 2.5. Bioinformatic Analysis

Raw sequencing reads, obtained from the Illumina platform, were then merged by using FLASH software (Version 1.2.7) [19] and filtered using QIIME software (Version 1.7), with the default parameter setting of ‘split_libraries_fastq.py’ script [20,21]. All quality filtered sequencing reads were then clustered into operational taxonomic units (OTUs) with a threshold of 97% sequence similarity, by utilizing UPARSE software (Version 7.0) [22]. The representative sequence (most abundant) for each bacterial OTU was then annotated by utilizing the GreenGene Database [23] and Human Oral Microbiome Database (HOMD) [24]. The least total sequences number was 30,070 in this study. The total reads of each sample was normalized to 30,070 sequences/sample, and the OTUs abundance information was normalized correspondingly for further analysis.

Based on these annotated and normalized output data, different statistical methods were used to interpret the similarities of diverse data sets, or to plot the correlation network among the salivary microbiota. First, community diversity estimators including Shannon and Simpson indexes were calculated by R software (Version 3.2.5) with vegan package. Second, the multiple response permutation procedure (MRPP) and analysis of similarity (Anosim) were employed to compare the statistical differences within and between subjects in salivary microbiota profiles, by using R software with vegan package [23]. Third, principal component analysis (PCA) was applied to evaluate and visualize the differences of samples in OTU-level complexity, by using R software with mixOmics package. Next, the correlations among the OTUs with relative abundance over 0.1% of each subject were calculated, based upon Pearson’s correlation coefficients, by using R software with Hmisc package. The strong connections (|r| > 0.9, *p* < 0.05) were further imported into Gephi software (Version 0.8.2), so as to generate correlation networks of these predominant microbiota [25]. The nodes (OTUs) with high strong connection numbers were defined as the “hub microbiota”, which were likely to be more connected to other nodes when compared to non-hub nodes [26,27]. Moreover, the relative abundance of the hub microbiota was further visualized into heatmaps, by utilizing R software with pheatmap package. Hierarchical clustering of the columns (samples) was further calculated based on Euclidean distance and ward.D method, and indicated on the heatmaps. Lastly, in order to identify the shared and unique hub salivary microbiome of these three subjects, a Venn diagram was built according to the method as descripted by Heberle et al. [28].

Other data are expressed as mean ± SD. Furthermore, the statistical significance among different data sets was analyzed by Student’s t-test or Duncan’s multiple range test using SPSS software (Version 19.0.0), while the significance threshold was established at 0.05.

## 3. Results

### 3.1. Phenolic Profile of Oolong Tea Infusion

The total polyphenols content and phenolic profile of the oolong tea infusion used in this study were determined, and the results indicated that the total polyphenol content of the tea infusion was 2.83 ± 0.02 g/L. Following untargeted UHPLC Q-TOF-MS approach, the phenolic constituents present in the tea infusion were further analyzed. Table 1 gives the MS characteristics and tentative identification of each chromatographic peak. These chromatographic peaks, along with their proposed chemical structure, are depicted in Figure S1. In summary, 33 constituents were tentatively identified from the tea infusion, including 2 alkaloids, 7 flavan-3-ols, 7 organic acids and esters, 4 proanthocyanidins, 11 flavonoid glycosides, 1 theaflavin, and 1 amino acid. Of the total chromatographic peak areas, caffeine (peak 16), epigallocatechin (peak 13), epicatechin (peak 20), gallic acid (peak 5), and caffeoyl-hexoside (peak 1) were the most abundant constituents.

### 3.2. Overall Salivary Bacterial Structure

The salivary bacterial components of the three subjects during the 12-week experimental period were investigated and evaluated using the Illumina HiSeq sequencing analysis. A total of 1,983,489 (average length = 425 bp) quality filtered sequencing reads corresponding to the V3–V4 region of bacterial 16S rRNA genes were obtained. Good’s coverage estimation values were within the range of 99.8%–100%, which indicated adequate sequence coverage to reliably describe the full bacterial communities present in all the samples. All sequences were clustered into 189 to 458 OTUs with a 97% similarity level for each sample. The summary of the sequencing results is listed in Table S1.

**Figure 1:**
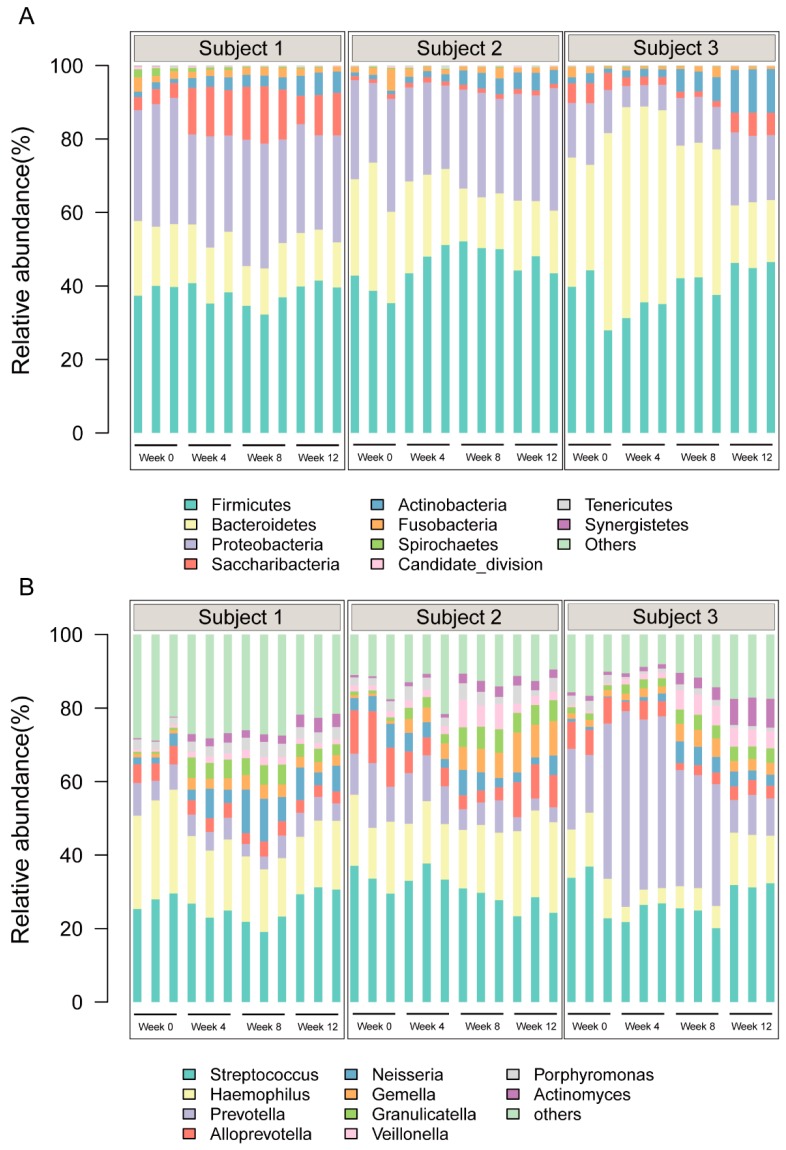
Relative abundances of the most abundant phyla and genera in each salivary sample in the (**A**) phylum level and (**B**) genus level.

After the taxonomic assignment, these sequences were then annotated into 25 phyla and 260 genera. At the phylum level, *Firmicutes* (41.04%), *Bacteroidetes* (24.23%), and *Proteobacteria* (23.31%) comprised the majority of OTUs (88.59%). While at the genus level, *Streptococcus* (28.24%), *Haemophilus* (15.97%), *Prevotella* (14.64%), *Alloprevotella* (5.27%), and *Neisseria* (4.21%) were the most prevalent bacterial taxa throughout the three subjects, which in totality accounted for 69.05% of all salivary bacteria. The relative abundance of these bacterial taxa at the phylum level and genus level are presented in Figure 1. These findings were generally in line with the findings of Belstrøm et al., which indicated the five most predominant genera identified were *Streptococcus*, *Haemophilus*, *Prevotella*, *Rothia*, and *Neisseria*, accounting for around 50% of the identified OTUs [29].

### 3.3. Comparisons of Salivary Bacterial Communities

Based on the relative abundance of all the OTUs, salivary bacterial community diversity (expressed by the Shannon and Simpson indexes) was investigated first, and the results are shown in Table 2. Compared with baseline (week 0), after eight weeks of tea consumption, a remarkable reduction in the community diversity was noticed across the three subjects, with the exception of the Shannon index of subject 3.

In order to adequately compare the homogeneity of salivary bacterial communities among the three subjects, MRPP and Anosim tests were then performed. In the pairwise comparisons, positive delta values from MRPP tests and R values from Anosim tests were observed, which indicated a higher similarity within the groups (Table 3). Thus, diversities of salivary microbiota among individuals were much larger than the variation within individuals over the course of tea consumption.

**Figure 2:**
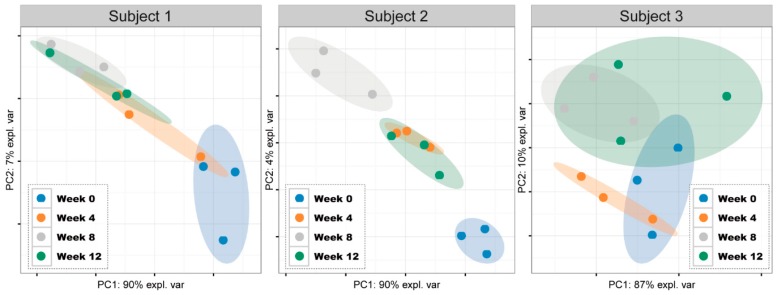
Principal component analysis (PCA) score plots based on the relative abundance of all operational taxonomic units (OTUs) of each subject.

The general profiles of salivary microbiota of each individual subject at different sampling times were further compared with PCA (Figure 2). For subject 1, the salivary bacterial communities in the baseline period were separated from the tea intervention and follow-up period, while in the follow-up period bacterial communities gathered with those in the tea consumption period. For subject 2, clear distinctions in the bacterial communities were discovered between week 0 and the other experimental periods, while the bacterial communities in week 12 and week 4 were overlapped. In the case of subject 3, relatively higher similarities were found among the different treatment periods, which might suggest a slighter or lower impact of tea consumption on the salivary microbiota.

### 3.4. Correlation Networks of Salivary Microbiota

**Figure 3:**
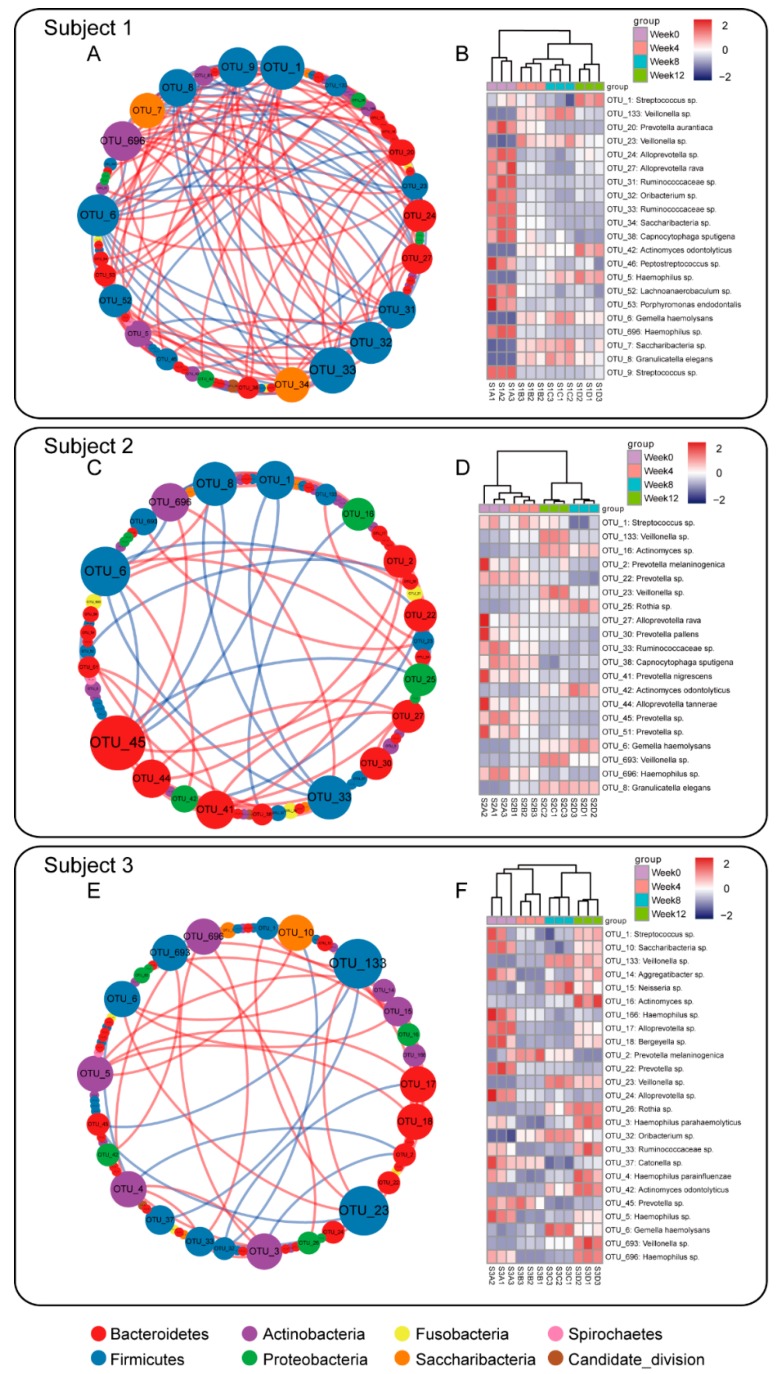
Correlation networks of the predominant salivary microbiota (**A**,**C**,**E**) and heatmaps of the hub salivary microbiota (**B**,**D**,**F**) in each subject. In correlation networks, each node represents an OTU; the color of nodes indicates the phylum information; the size of nodes represents the number of linkages; lines between nodes represent a strong correlation between these two OTUs (|r| > 0.9 and *p* < 0.05, Pearson’s correlation); red line represents a positive correlation and blue line represents a negative correlation. The nodes with high strong connection numbers were selected as the “hub microbiota” and their dynamic shifts of relative abundance were further depicted on heatmaps. The color of the data matrix in heatmaps corresponds to the normalized relative abundance of the OTUs; the color bar on the top right indicates the scale.

Based on the Illumina sequencing results, 67 OTUs were defined as the predominant salivary microbiota of the three subjects, with relative abundance over 0.1%. Pearson’s correlations were calculated among the predominant salivary microbiota of each subject, and the strong connections (|r| > 0.9 and *p* < 0.05) were further visualized as networks (Figure 3A,C,E). When comparing the networks of the three subjects, subject 1 had the most complicated co-occurrence patterns of salivary bacteria, with a total strong connection number of 128. For subjects 2 and 3, the strong connection numbers were 49 and 41, respectively.

### 3.5. Hub Salivary Microbiota Identification

The size of each node in the network represents the number of strong connections with other nodes. Thus, the OTUs with a larger node size were identified as the hub salivary microbiota of each subject, which had more connections with other bacteria. In this study, approximately 20 hub OTUs from each subject were intentionally selected. In particular, for subject 1, 21 OTUs were defined as the hub microbiota (Figure 3A). The relative abundance changes of these bacteria were further visualized as a heatmap plot (Figure 3B). Of these, 8 OTUs (OTU 133, 23, 42, 5, 6, 7, 8, and 9) increased after tea intervention, while the remaining 13 OTUs decreased; moreover, OTU 1, 42, and 5 increased during the follow-up period (week 12). For subjects 2 and 3, 20 and 25 OTUs were identified as hub microbiota (Figure 3C,E). The successions of these hub salivary microbiota during the 12-week experimental period are illustrated in Figure 3D,F.

**Figure 4:**
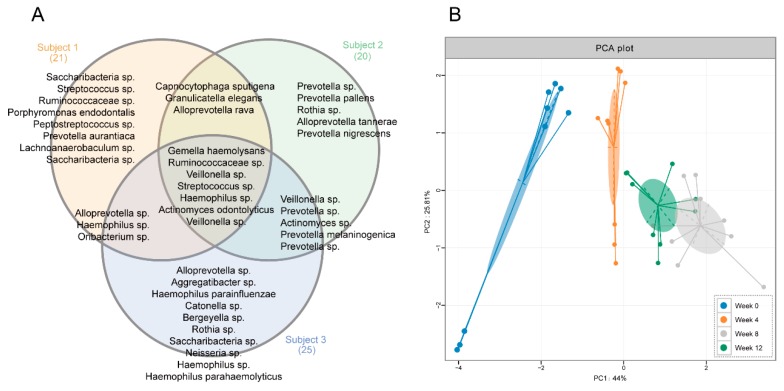
(**A**) The Venn diagram of the hub salivary microbiota in each subject. (**B**) PCA score plots based on the relative abundance of the shared hub microbiota across the three subjects.

**Figure 5:**
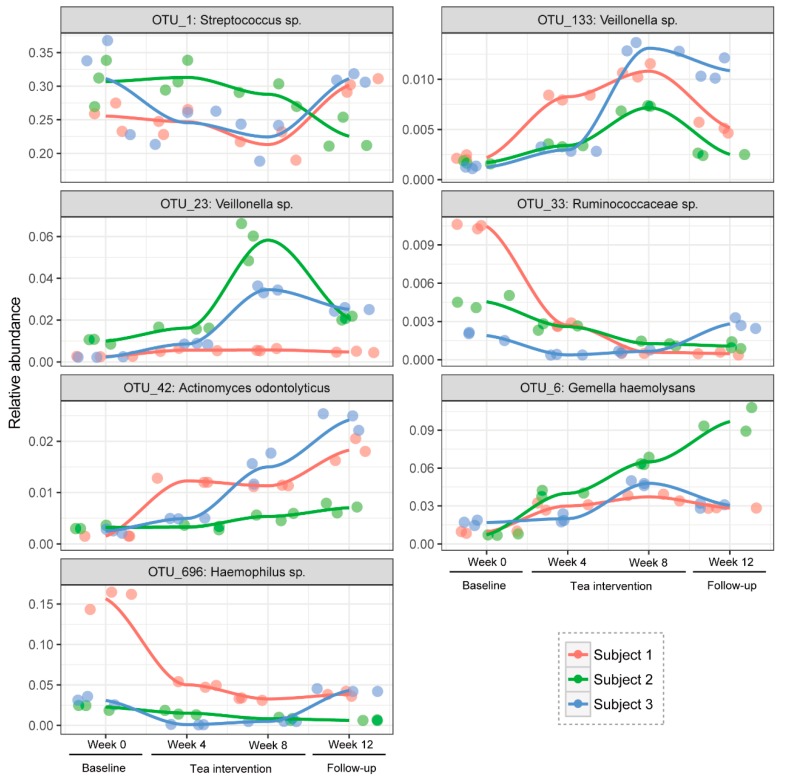
The temporal shifts of the shared hub salivary microbiota during the 12-week experimental period.

Through a Venn diagram, seven OTUs, including OTU_1 (*Streptococcus* sp.), OTU_133 (*Veillonella* sp.), OTU_23 (*Veillonella* sp.), OTU_33 (*Ruminococcaceae* sp.), OTU_42 (*Actinomyces odontolyticus*), OTU_6 (*Gemella haemolysans*), and OTU_696 (*Haemophilus* sp.), were identified as the shared hub microbiota of the three subjects (Figure 4A). The unique hub salivary microbiome is also shown in Figure 4A. Based on the relative abundance of these shared hub bacteria during the entire experimental period for the three subjects, a PCA plot was further depicted (Figure 4B). A clear separation of the baseline period (week 0) from other score points was observed, which revealed a significant change with regard to these seven OTUs which occurred after tea infusion drinking. The PCA score plots of week 4 and week 8 were gathered into two discrete clusters, which indicated a time-dependent response of these bacteria to tea drinking. For week 12, this cluster was in-between those of week 4 and week 8, indicating a relatively similar bacterial profile pattern in the follow-up period with tea treatment. The temporal shifts of these seven shared hub salivary microbiota during the 12-week experimental period are reflected in Figure 5. In general, compared with the baseline period, in week 4, *Ruminococcaceae* sp. (OTU_33) and *Haemophilus* sp. (OTU_696) were suppressed significantly (*p* < 0.05), while *Veillonella* sp. (OTU_133), *Actinomyces odontolyticus* (OTU_42), and *Gemella haemolysans* (OTU_6) were promoted significantly (*p* < 0.05). After eight weeks of tea consumption, *Streptococcus* sp. (OTU_1), *Ruminococcaceae* sp. (OTU_33), and *Haemophilus* sp. (OTU_696) were suppressed significantly (*p* < 0.05), while *Veillonella* spp. (OTU_133 and OTU_23), *Actinomyces odontolyticus* (OTU_42), and *Gemella haemolysans* (OTU_6) were promoted significantly (*p* < 0.05). In the follow-up period, only *Streptococcus* sp. (OTU_1) was return to its initial level (*p* > 0.05).

## 4. Discussion

In the present study, an oolong tea infusion containing a total of 2.83 ± 0.02 g/L polyphenols, including catechin, epicatechin, epigallocatechin gallate, and at least 30 other components, was used to evaluate its salivary microbiota modification effect. Three subjects were required to consume 1.0 L of tea infusions daily, which equaled approximately 52 mg/kg body weight of tea polyphenols. The preparation method and intake amount of tea infusions followed the general tea drinking habits of individuals in the southern part of China, which would provide a more pragmatic and appropriate insight. Under this consumption amount, diverse responds of salivary microbiota were observed among the three subjects.

Following the Illumina high-throughput sequencing, a highly diverse salivary bacterial community was observed. A total of 8801 OTUs with a 97% similarity level was identified from the 36 saliva samples and annotated into 25 phyla and 260 genera. In addition to the complexity, a high inter-individual variation in salivary microbiota was also discovered. It was revealed that the salivary microbial communities within the three subjects were significantly distinct from each other, exhibiting host-specific microbiota profiles; their overall collective responses to tea consumption also varied among each participant. The positive delta values from MRPP tests and R values from Anosim tests indicated the differences of salivary microbiota profiles among subjects were far more significant than that among different time points within one subject (Table 3). When all 36 salivary microbiota data sets from the three subjects were depicted into one PCA plot, no clear cluster was observed (Figure S2). The host-specific salivary microbiota were also confirmed by the distinct correlation networks of each participant. These results were consistent with the findings of Belstrøm et al., since the authors confirmed that the five individuals in their study had a personalized salivary bacterial fingerprint [29]. Hall et al. also stated that the oral bacterial community fingerprint varied from person to person in their study [17]. Thus, in order to minimize the inter-individual variations, the data sets from different subjects were analyzed separately, otherwise the effect of tea may be obscured by the inter-individual variations, as shown in Figure S2.

In general, it was revealed that oolong tea consumption led to a profound reduction in diversity of the salivary bacterial communities of subject 1 and subject 2. Takeshita et al. stated in their population-based study that good oral health was associated with a lower phylogenetic diversity of the salivary microbiome [5]. Moreover, Vestman et al. reported that the diversity of the tooth biofilm samples was reduced after probiotics supplementation [30]. The increase of diversity of gut microbiota is normally associated with better gut health conditions, such as through the extension of the functional genes for facilitation of the absorption of nutrients and energy, or for appropriate development of immunity. In contrast to the commensal microbiota residents in the intestinal tract, which typically live in harmony with the host, the oral microbiota is responsible for the two most common diseases, including dental caries and periodontal diseases [31]. The increase of diversity in salivary microbiota may be associated with the flourish of dental plaque which resulted from the accumulation of attached bacteria, and thus increase the risk of dental caries and periodontal diseases; while, the decrease in taxonomic diversity in saliva may indicate the shrinking of bacterial communities in dental plaque biofilms, and thus lead to healthier oral ecological conditions. However, for subject 3, the decrease of the salivary microbial community diversity was not significant, except for Simpson index of week 8, which might indicate a lower modulation effect of tea on subject 3. Furthermore, according to PCA, significant overall shifts of salivary microbiota composition were noted in subjects 1 and 2. However, in the case of subject 3, a higher variation was discovered amongst different sampling time points, which may also suggest a lower effectiveness of tea consumption upon the salivary microbiota of subject 3.

The oral cavity, as the portal of entry to the gastrointestinal tract, is one of the most complex microbial colony sites within the human body [32]. In order to better understand the complex ecologic system, a correlation network was employed in this study to simplify and visualize the co-occurrence patterns of salivary bacteria. The bacteria taxa with robust connections with other salivary bacteria were defined as “hub salivary bacteria”. Subsequently, via a heatmap plot, the temporal dynamic of each individual hub salivary bacteria was clearly presented. Afterwards, through a Venn diagram, the shared hub microbiota across the three subjects were further identified. Separated correlation network analysis revealed the detailed influence of tea consumption on the salivary microbiota composition within the same contactable environment. Thus, it minimized the inter-individual variations between subjects. While, an additional Venn diagram further helped in seeking the common influences of tea consumption.

In particular, seven shared hub OTUs across the three subjects were identified from the highly complex and personalized oral ecosystem. Notably, OTU_1 (*Streptococcus* sp.), as the most predominant taxon, also acted as a shared hub microbiota and favorably interacted with other oral bacteria. Due to the biofilm formation and acid production ability of *Streptococcus*, multiple members of this genus, including *Streptococcus mutans, Streptococcus sobrinus, Streptococcus salivarius, Streptococcus constellatus*, and *Streptococcus parasanguinis*, were considered as opportunistic pathogens [33]. With regard to the shifts of *Streptococcus* sp., a significant decrease (−16.94%, *p* = 0.035) was found after eight weeks of tea consumption. Therefore, a *Streptococcus* inhibitory effect of tea was observed in this study, and the effect may assist in the prevention of dental caries. There is a preponderance of evidence to support the beneficial role of tea in protecting against this oral pathogen. Narotzki et al. reviewed the clinical and biological studies regarding the correlation between green tea and oral health and concluded that green tea may reduce dental caries through bacterial growth repression and enzyme activity inhibition [14]. With the exception of green tea, it has also been reported that black tea extracts could inhibit *S. mutans* adhesion in vitro [34]. Kawarai et al. compared the *S. mutans* biofilm formation inhibitory effect of Assam tea (a black tea variety) and green tea and ascertained that Assam tea exhibited a stronger biofilm inhibition activity than green tea [35]. The inhibitory activity of specific teas against oral pathogens are commonly attributed to the phenolic components within the tea [14].

Similar inhibitory effects were also observed on OTU_33 (*Ruminococcaceae* sp.) and OTU_696 (*Haemophilus* sp.), both of which were also hub microbiota across the three subjects. *Haemophilus* are a common bacteria which inhabit the mouth, vagina, and intestinal tract. The genus includes commensal organisms, along with some pathogenic species such as *H. influenzae* and *H. ducreyi*. The inhibitory effect of tea on *Haemophilus* may also reduce the risk of infection. *Ruminococcaceae*, one of the most typical gut microbiotas, can be found in significant numbers in the intestines of humans. However, the biological meaning regarding the depletion of this bacterium induced by tea drinking was not clear.

Along with oolong tea consumption, a significant elevation of OTU_133 (*Veillonella* sp.), OTU_23 (*Veillonella* sp.), OTU_42 (*Actinomyces odontolyticus*), and OTU_6 (*Gemella haemolysans*), which were all demonstrated as robust network nodes across the three subjects, was observed in this study. Lim et al. illustrated a significant negative association between *Haemophilus* and *Veillonella* [36], which was consistent with our findings. Furthermore, it was reported that the establishment of some certain oral commensals was linked to oral health, such as the bacterial species belonging to *Neisseria*, *Veillonella*, and *Actinomyces* [37], although details regarding the exact mechanisms are not yet available. Moreover, the elevated effect on these four hub bacteria continued throughout the follow-up period, which demonstrated the sustained effect of tea drinking.

With regard to the mechanisms behind the modification effect of tea on salivary microbiota, several hypotheses have been invoked to account for this particular effect: (i) tea polyphenols possess antimicrobial properties, which are believed to aid in the inhibition of certain bacteria, including some pathogens [13,14]; (ii) tea polyphenols as antioxidants may alleviate oral oxidative stress and inflammation, which may further impact the oral immune system and induce a drift of the bacterial community [14]; (iii) tea polyphenols can precipitate salivary proteins and inhibit the activity of salivary alpha-amylase, and thus, induce the decrease of fermentation of carbohydrates involved in caries formation [38]. However, the precise mechanism is still ambiguous, resulting in the necessity for further studies. Numerous epidemiologic studies and clinical trials have validated that regular tea consumption could reduce the risk of cardiovascular disease, including coronary heart disease, stroke, and peripheral arterial disease [39]. Recent studies show a correlation between periodontal disease and cardiovascular disease [40,41]. Thus, from the perspective of alleviating systematic inflammatory and immunological processes, explicating the underlying mechanisms (e.g., to link the levels of endogenous mediators, such as endothelin [42] and vitamins [9] of tea consumption may open an innovative avenue toward the development of new antibiotics with good safety and tolerability margin.

It was also acknowledged that the inadequate number of subjects in this study might limit the statistical analysis. As explained previously, using limited subjects and following the time course of each individual may help to minimize the inter-individual variations. However, further studies with larger sample sizes are warranted to validate these findings.

## 5. Conclusions

In summary, using three healthy adult volunteers as our subjects, our study demonstrated that a daily consumption of 1.0 L oolong tea for eight weeks caused a reduction in bacterial community diversity, as well as the disturbance of hub salivary bacterium with strong connections to other salivary microbiota. Additionally, it was also noticed that large inter-individual variations were found, implying diverse responses to oolong tea consumption may exist among subjects. Larger sample sizes and more in-depth mechanism studies are necessary to further clarify and elucidate the physiological relevance of the shifts of salivary microbiota to the oral health of the host.

## Figures and Tables

**Table 1:** The phenolic profiles of the oolong tea infusion.

Peak No. ^a^	*t*_R_ (Min)	Tentative Identification	Chemical Formula	[M-H]^−^ (m/z)		
				Measured Mass (Da)	Theoretical Exact Mass (Da)	Mass Accuracy (ppm)
1	1.05	Caffeoyl-hexoside	C_15_H_18_O_9_	341.0875	341.0873	0.58
2	1.40	L-Theanine	C_7_H_14_N_2_O_3_	173.0931	173.0927	2.53
3	1.97	Epigallocatechin-glucuronide	C_21_H_22_O_13_	481.0991	481.0983	1.74
4	2.49	Theasinensin C	C_30_H_26_O_14_	609.1235	609.1245	−1.60
5	2.74	Gallic acid	C_7_H_6_O_5_	169.0140	169.0137	1.52
6	2.92	Theogallin	C_14_H_16_O_10_	343.0665	343.0666	−0.19
7	3.80	Theobromine ^b^	C_7_H_8_N_4_O_2_	181.0736	181.0725	6.04
8	3.84	Gallocatechin	C_15_H_14_O_7_	305.0662	305.0662	0.09
9	4.37	Theasinensin B	C_37_H_30_O_18_	761.1348	761.1354	−0.83
10	4.41	Digalloyl-hexoside	C_20_H_20_O_14_	483.0758	483.0775	−3.57
11	4.54	O-Methylgallic acid	C_8_H_8_O_5_	183.0295	183.0294	0.58
12	4.81	Theacitrin A	C_37_H_28_O_18_	759.1196	759.1198	−0.24
13	4.91	Epigallocatechin	C_15_H_14_O_7_	305.0689	305.0662	8.94
14	5.16	*p*-Coumaroylquinic acid	C_16_H_18_O_8_	337.0923	337.0924	−0.26
15	5.36	Catechin	C_15_H_14_O_6_	289.0718	289.0713	1.87
16	5.60	Caffeine ^b^	C_8_H_10_N_4_O_2_	195.0888	195.0882	3.30
17	5.68	Procyanidin	C_30_H_26_O_12_	577.1356	577.1346	1.65
18	5.79	Epicatechin-epicatechin	C_30_H_26_O_12_	577.1356	577.1346	1.65
19	6.14	*p*-Coumaroylquinic acid	C_16_H_18_O_8_	337.0923	337.0924	−0.26
20	6.23	Epicatechin	C_15_H_14_O_6_	289.0734	289.0713	7.41
21	6.34	Epigallocatechin gallate	C_22_H_18_O_11_	457.0777	457.0771	1.24
22	6.41	*p*-Coumaroylquinic acid	C_16_H_18_O_8_	337.0918	337.0924	−1.74
23	6.68	Gallocatechin gallate	C_22_H_18_O_11_	457.0773	457.0771	0.37
24	6.92	Theaflavin	C_29_H_24_O_12_	563.1199	563.1190	1.60
25	7.01	Myricetin-hexoside	C_21_H_20_O_13_	479.0827	479.0826	0.19
26	7.11	Myricetin-hexoside	C_21_H_20_O_13_	479.0825	479.0826	−0.23
27	7.21	Quercetin-hexosyl-hexosyl-deoxyhexoside	C_33_H_40_O_21_	771.1986	771.1984	0.22
28	7.36	Quercetin-hexosyl-hexosyl-deoxyhexoside	C_33_H_40_O_21_	771.1982	771.1984	−0.30
29	7.62	Kaempferol-deoxyhexosyl-deoxyhexoside	C_27_H_30_O_14_	577.1555	577.1558	−0.48
30	7.72	Kaempferol-hexosyl-hexosyl-deoxyhexoside	C_33_H_40_O_20_	755.2029	755.2035	−0.81
31	8.00	Kaempferol-hexosyl-hexosyl-deoxyhexoside	C_33_H_40_O_20_	755.2048	755.2035	1.70
32	8.43	Kaempferol-hexosyl-hexoside	C_27_H_30_O_15_	593.1508	593.1507	0.18
33	8.78	Kaempferol-hexoside	C_21_H_20_O_11_	447.0927	447.0928	−0.18

^a^ Peaks were assigned from the chromatograms in Figure S1; ^b^ [M+H]^+^ mode.

**Table 2:** The temporal changes of the salivary microbial community diversity in each subject.

	Baseline	Tea Intervention		Follow-Up
	Week 0	Week 4	Week 8	Week 12
**Subject 1**				
Shannon	5.28 ± 0.41 ^a^	4.68 ± 0.27 ^ab^	4.00 ± 0.39 ^b^	4.17 ± 0.40 ^b^
Simpson	0.94 ± 0.02 ^a^	0.89 ± 0.03 ^ab^	0.81 ± 0.04 ^b^	0.84 ± 0.06 ^b^
**Subject 2**				
Shannon	4.79 ± 0.58 ^a^	4.63 ± 0.22 ^ab^	4.02 ± 0.27 ^b^	4.37 ± 0.13 ^ab^
Simpson	0.91 ± 0.06 ^a^	0.88 ± 0.06 ^a^	0.83 ± 0.05 ^b^	0.90 ± 0.02 ^a^
**Subject 3**				
Shannon	3.99 ± 0.57 ^a^	3.93 ± 0.27 ^a^	3.90 ± 0.17 ^a^	4.07 ± 0.65 ^a^
Simpson	0.85 ± 0.10 ^a^	0.83 ± 0.03 ^a^	0.80 ± 0.03 ^b^	0.83 ± 0.09 ^a^

Values are expressed as the mean ± SD (*n* = 3). Means with different superscript letters (a, b) within a row suggest significant differences (*p* < 0.05); means with the same superscript letters (a, b) within a row suggest the differences are not significant (*p* ≥ 0.05), as determined by Duncan’s multiple range test.

**Table 3:** Summary of multiple response permutation procedure (MRPP) and analysis of similarity (Anosim) tests between each subject.

Compared Data Sets	MRPP		Anosim	
	Delta	*p*-Value	R	*p*-Value
Subject 1 vs. Subject 2	0.1969	0.001	0.7105	0.001
Subject 1 vs. Subject 3	0.1479	0.001	0.4886	0.001
Subject 2 vs. Subject 3	0.1919	0.001	0.7562	0.001
Subject 1 vs. Subject 2 vs. Subject 3	0.2227	0.001	0.6482	0.001

## Supplementary Materials

The following are available online at https://www.mdpi.com/2072-6643/12/4/966/s1, Figure S1: Chromatograms obtained from oolong tea infusion, using UHPLC Q-TOF-MS/MS in negative and positive ion modes, Figure S2: PCA score plot based on the relative abundance of all OTUs of the three subjects, Table S1: Summary of the sequencing results of all salivary samples.
