# Supplementary material for: Salivary Microbiota Shifts under Sustained Consumption of Oolong Tea in Healthy Adults
Source: Nutrients. 2020 Mar 31;12(4):966. doi: 10.3390/nu12040966 (PMC7230163; doi:10.3390/nu12040966)
Supplement: Supplementary file 1 [file nutrients-12-00966-s001.zip › Figure S1.pdf]

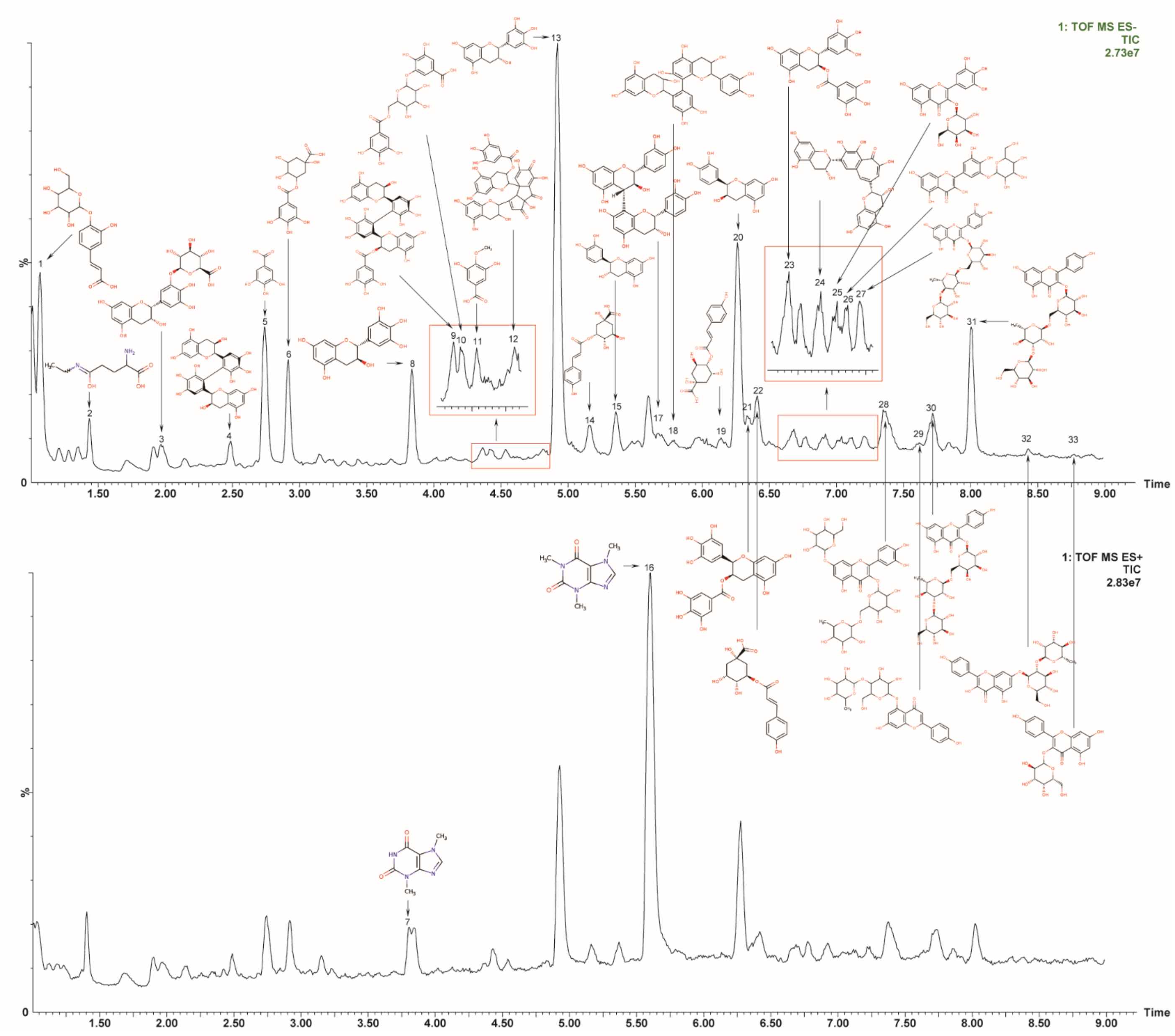

Figure S1. Chromatograms obtained from oolong tea infusion, using UHPLC-Q-TOF-MS/MS in negative and positive ion modes.
