# Supplementary material for: Salivary Microbiota Shifts under Sustained Consumption of Oolong Tea in Healthy Adults
Source: Nutrients. 2020 Mar 31;12(4):966. doi: 10.3390/nu12040966 (PMC7230163; doi:10.3390/nu12040966)
Supplement: Supplementary file 1 [file nutrients-12-00966-s001.zip › Table S1.docx]

**Table S1.** Summary of the sequencing results of all salivary samples

| **Sample** | **Total Tags^a^** | **Taxon Tags^b^** | **Average Length (bp)** | **OTU Number** | **Good's Coverage** |
| --- | --- | --- | --- | --- | --- |
| Subject 1 | | | | | |
| S1A1 | 65186 | 64705 | 421 | 267 | 99.9% |
| S1A2 | 53507 | 52346 | 423 | 243 | 99.9% |
| S1A3 | 58481 | 58254 | 424 | 256 | 99.9% |
| S1B1 | 50535 | 50218 | 424 | 255 | 99.9% |
| S1B2 | 52585 | 52287 | 423 | 264 | 99.9% |
| S1B3 | 55710 | 55464 | 424 | 278 | 99.8% |
| S1C1 | 50190 | 49684 | 427 | 249 | 99.9% |
| S1C2 | 51982 | 51392 | 427 | 221 | 99.9% |
| S1C3 | 40568 | 40354 | 426 | 247 | 99.9% |
| S1D1 | 61354 | 60794 | 425 | 260 | 99.9% |
| S1D2 | 57478 | 56903 | 426 | 239 | 99.9% |
| S1D3 | 54803 | 53851 | 424 | 236 | 99.9% |
| Subject 2 | | | | | |
| S2A1 | 65384 | 65160 | 427 | 224 | 99.9% |
| S2A2 | 56113 | 55887 | 425 | 236 | 99.9% |
| S2A3 | 60897 | 60647 | 426 | 306 | 99.8% |
| S2B1 | 50818 | 50642 | 426 | 252 | 99.9% |
| S2B2 | 30138 | 30070 | 425 | 201 | 100.0% |
| S2B3 | 49400 | 48890 | 427 | 458 | 99.8% |
| S2C1 | 55950 | 55817 | 426 | 192 | 99.9% |
| S2C2 | 54763 | 54638 | 426 | 209 | 99.9% |
| S2C3 | 56595 | 56408 | 427 | 273 | 99.8% |
| S2D1 | 53737 | 53517 | 424 | 235 | 99.9% |
| S2D2 | 60492 | 60275 | 426 | 322 | 99.8% |
| S2D3 | 59558 | 59418 | 423 | 222 | 99.9% |
| Subject 3 | | | | | |
| S3A1 | 52639 | 52348 | 422 | 230 | 99.9% |
| S3A2 | 63888 | 63368 | 424 | 243 | 99.9% |
| S3A3 | 60488 | 60093 | 425 | 195 | 99.9% |
| S3B1 | 63893 | 63564 | 426 | 247 | 99.9% |
| S3B2 | 45346 | 45137 | 426 | 209 | 99.9% |
| S3B3 | 50683 | 50463 | 424 | 235 | 99.9% |
| S3C1 | 54309 | 54099 | 425 | 189 | 99.9% |
| S3C2 | 62980 | 62820 | 426 | 210 | 99.9% |
| S3C3 | 66433 | 66221 | 424 | 260 | 99.8% |
| S3D1 | 51468 | 51323 | 427 | 220 | 99.9% |
| S3D2 | 65899 | 65747 | 427 | 206 | 99.9% |
| S3D3 | 50823 | 50685 | 425 | 212 | 99.9% |

^a^ Total tags were the splicing sequences; ^b^ Taxon tags were the high-quality clean tags obtained by quality filtering.
