# Supplementary figures and images for: Salivary Microbiota Shifts under Sustained Consumption of Oolong Tea in Healthy Adults
Source: Nutrients. 2020 Mar 31;12(4):966. doi: 10.3390/nu12040966 (PMC7230163; doi:10.3390/nu12040966)

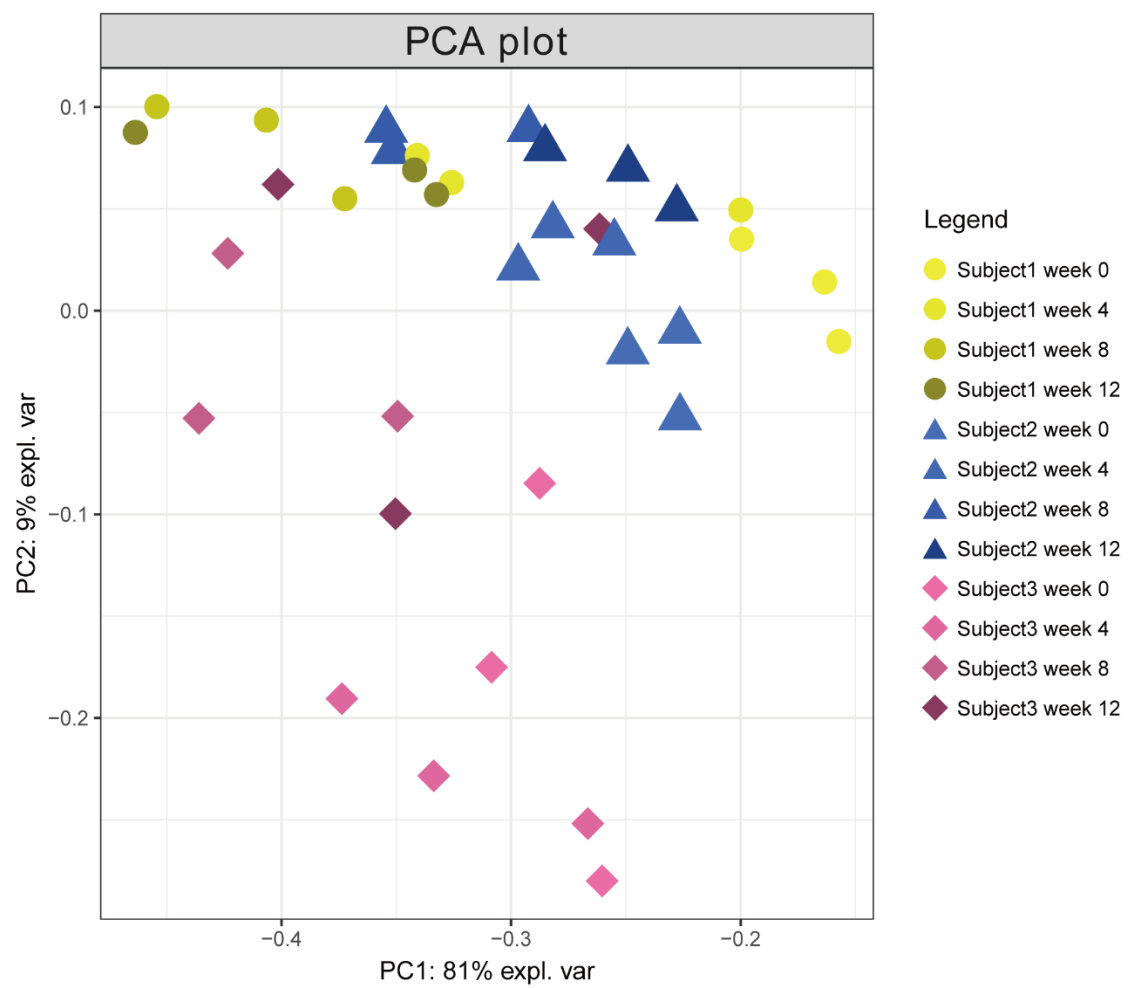

**Figure S2.** PCA score plot based on the relative abundance of all OTUs of the three subjects.

Supplement: Supplementary file 1 [file nutrients-12-00966-s001.zip › Figure S2.pdf]
